# Investigating the Links Between Performers’ Self-Compassion, Mental Toughness and Their Social Environment: A Semi-Systematic Review

**DOI:** 10.3389/fpsyg.2022.887099

**Published:** 2022-07-14

**Authors:** Celine Kosirnik, Roberta Antonini Philippe, Valentino Pomini

**Affiliations:** ^1^Institute of Sports Sciences, University of Lausanne, Lausanne, Switzerland; ^2^Institute of Psychology, University of Lausanne, Lausanne, Switzerland

**Keywords:** self-compassion, mental toughness, social environment, performing arts, sport

## Abstract

Being mentally tough while evaluating oneself in a compassionate way is still a difficult path for performers. Self-compassion, characterized by the ability to be kind to oneself, to see one’s experiences as part of the larger human experience and have a balanced awareness to one’s emotions and thoughts, was recently studied as a stepping stone to performance optimization and personal development. Despite a mistrust of this concept in the sports world, various studies show its benefits within athletes. A major question remains the environment that fosters or hinders the development of self-compassion: when role models extend compassionate attitudes, does it allow performers to respond in more self-compassionate ways? The relationship between self-compassion, mental toughness, and social environment is still unclear and is an important direction for future research within performers. This semi-systematic literature review aims at proposing an overview of the state of the art regarding self-compassion, mental toughness, and the influence of performer’s, and social environments. Sixteen studies were retrieved. We conclude that the number of multi-day intervention programs and longitudinal studies should be increased. The studies should also consider assessing the specific aspects of performance culture and settings. In addition, overall performance-specific measures could be developed to assess general levels of self-compassion. The development of a theoretical framework explaining how self-compassion affects a performer, the role of their entourage and its link to other psychological resources, such as mental toughness, could help to better understand this concept.

## Introduction

Performance, in different manner, is often at the heart of our daily activities, whether at work (e.g., Van Daalen et al., 2009) or in our personal lives (e.g., DeGroot and Vik, 2021). It is even more present in the domains of sport and performing arts. Promoting athletes’ and, more broadly, performers’ (i.e., artists, musicians, dancers) mental health is a significant area of scientific research in sport and performance sciences to enable high-performance over time (Donohue et al., 2018; Van Slingerland et al., 2019). How can athletes and performers be accompanied in developing their mental health and personal resources to cope with this challenging world and maintain high-performance level? What key approaches could help to nurture successful, resilient performers and consider their mental health and well-being?

Athletes must often, if not constantly, give their best and do their utmost to achieve high performances. Physical preparation, practicing their sporting skills, nutritional follow-up, and the planning and management of their training sessions are all frequently put forward as essential elements in their struggles to achieve great things (Baker et al., 2003; Macquet and Skalej, 2015). Over the last few decades, but especially in the last 10 years, mental training and sport psychology have become integral parts of an athlete’s preparation and support. Mental training and sport psychology aim to prepare athletes to handle the stress, pressure, and emotions they are confronted to. It helps them learn about themselves to better cope with challenges (Ong and Harwood, 2018).

The mental toughness of high-level performers is often put forward when discussing their performances (Bauman, 2016; Gucciardi and Hanton, 2016; Morais and Gomes, 2019). Athletes or their relatives frequently turn to sports psychologists or mental trainers with the request to become mentally tough or develop this toughness (Gee et al., 2010). Indeed, mental toughness is often considered an essential personal characteristic of top performers, consisting of aspects of psychological individuality including positive cues resulting from personal resources (e.g., self-efficacy, optimism) (Gucciardi et al., 2017). However, mental toughness is often followed by harmful beliefs. High-performance environments foster conditions in which a performer’s personal resources, such as those encompassed by mental toughness (e.g., overcoming obstacles, perseverance), become so highly valued that athletes may be less likely or willing to seek help for mental health issues (Gucciardi et al., 2017). Furthermore, athletes’ mental toughness is influenced by their social environment and surroundings (Antonini Philippe et al., 2016; Bean et al., 2019). Research has investigated the mechanisms linking elements of coach-created environments to certain positive and negative indicators of athletes’ well-being and mental toughness (Gagne, 2003; Reinboth and Duda, 2004). Similarly, weak social support appears to be a significant risk factor in overall mental health (Rice et al., 2016).

Also, to perform at their best, performers not only have to remain mentally strong, but they need to understand the mistakes and difficulties they encounter to find solutions to move forward. Sport psychology research, to cope with difficulties, was interested, for example, in the concepts of resilience (e.g., Sorkkila et al., 2019), grit (e.g., Newland et al., 2020) or, more recently, self-compassion (Doorley et al., 2022). Self-compassion was showed to have significant impact on athletes’ motivation and capacity to bounce back after difficult times (e.g., injuries, failure). However, research in this field is relatively new; therefore, we still have to understand what influences and helps develop self-compassionate competencies.

Through a semi-systematic review, defined as a review methodology for “topics that have been conceptualized differently and studied by various groups of researchers within diverse disciplines […] and useful for detecting themes, or common issues within a specific research discipline” (Snyder, 2019), this article will investigate the concepts of mental toughness, self-compassion within performers and the links with their social environment. Mental toughness and self-compassion are also being studied in the world of performing arts and, as proven by some authors, bridges can be built between the world of sport, performing arts, music, and dance (Pecen et al., 2018). Therefore, the word *performers*, in this article, includes athletes, musicians and dancers.

### Mental Toughness, Self-Compassion, and the Social Environment: A Winning Combination for Performers’ Well-Being and Performances

Experiences of failure, leading to emotional distress, which are inherent to high-performance fields, can be detrimental to performers’ future performances and mental health. As previously mentioned, high-performance activities are often symbolized by courage, grit, and mental toughness (Baltzell et al., 2020). However, this mental toughness can have detrimental elements (Gucciardi et al., 2017). One emerging concept being studied and highlighted as a counterbalance and complement to mental toughness is self-compassion (Baltzell et al., 2020). Self-compassion, or treating oneself with kindness (Neff, 2003a), “can help performers deal with failure and can serve as a buffer against negative emotional psychological reactions” (Ceccarelli et al., 2019, p. 1). Neff examined the positive psychological functions of self-compassion and reported that self-compassionate individuals were more likely to think positively, have greater self-awareness, and make accurate self-appraisals (Neff et al., 2007; Neff, 2009; Neff and Germer, 2013). Self-compassion is defined by a supportive attitude toward oneself and includes being kind to oneself, common humanity, and mindfulness (Neff, 2003b). Common humanity is defined as seeing one’s experiences as part of the larger human experience rather than seeing them as separate and isolating (Baltzell, 2016). Mindfulness corresponds to the ability to take a step back from our overarching emotions and thoughts and ‘be in the moment.’ Researchers working in the field of sport psychology have begun to address this concept with athletes (Ferguson et al., 2015; Mosewich et al., 2019), finding links between self-compassion and performance-related factors. Athletes with relatively high levels of self-compassion perform better and cope more effectively with sport stressors (Baltzell, 2016). Athletes with higher levels of self-compassion have also been shown to be relatively more positive, more persistent, less ruminative, and more willing to take responsibility (Ferguson et al., 2015). Finally, they also seem to have relatively greater experience in self-discipline along with less body shame (and thus less body monitoring), less fear of failure, less fear of negative evaluations, and less social physical anxiety (Mosewich et al., 2011; Baltzell, 2016). However, some athletes have assumptions about self-compassion, such as that if they were more compassionate with themselves, they would lose their intrinsic motivation and feel sorry for themselves (Baltzell et al., 2020). When athletes are more compassionate with themselves, it is easier for them to ‘tolerate’ the (primarily negative) emotions they go through when faced with undesirable experiences in their sport (Reis et al., 2015). More research into this concept is therefore essential as it has enormous potential in improving well-being and performance. Furthermore, it is interesting to look at performers in a broader sense (including dancers and musicians) because self-compassion among these populations has received little attention to date (Tarasoff et al., 2017; Kveton-Bohnert, 2018; Johnson, 2020).

Moreover, in the last 20 years, different authors have been interested in how a performer’s social environment can affect their performances and well-being (Jowett and Poczwardowski, 2007; Antonini Philippe et al., 2011; Wachsmuth et al., 2020). When talking about the social environment’s influence, we assume the way performers perceive and appraise their environment, interactions and conditions often conveyed by coaches through the creation of a certain type of environment (e.g., autonomy supportive, compassionate). Studies have revealed that developing caring environment and compassion for self and others have a range of benefits on psychological processes (Neff, 2011; Jazaieri et al., 2013; Keltner et al., 2014) and social relationships (Penner et al., 2004; Crocker and Canevello, 2012; Neff and Beretvas, 2013; Neff and Pommier, 2013). Research in sport psychology has highlighted that when role models show an accepting attitude toward performers, regardless of outcomes, these positive interactions also encourage athletes to respond in more self-accepting ways (Ingstrup et al., 2017). A coach or a parent’s attitude can maximize a performer’s potential in terms of performance as well as toward their personal and psychological development (self-esteem, self-confidence, motivation). According to the study by Pecen et al. (2018), performers, among their many challenges, may also face difficulties from within their social environment, such as a bad or abusive coach, manipulative parents, dangerous advice, sexual harassment, or abuses of power and positions of influence. Coaches and parents are instrumental in creating social environments that can influence an athlete’s physical growth and, psychological and subjective well-being (Jowett and Cockerill, 2003; Blanchard et al., 2009; Bartholomew et al., 2011).

### Previous Literature Reviews on Self-Compassion, Physical Activity, and Sport

Previous literature reviews have focused on the concept of self-compassion within sport or physical activity in general. Röthlin et al. (2019) did a scoping review of the published evidence on self-compassion research in competitive sport. They answered the question about whether self-compassion is beneficial to competitive athletes. They were interested in the methodologies used in self-compassion studies. Overall, the review highlighted self-compassion’s benefits and revealed how it influenced well-being, reduced feelings of shame and self-criticism, and improved the ability to deal with failures. However, few of these studies investigated personal and social variables. Some highlighted that when athletes have the impression that they are supported socially (e.g., by teammates), self-compassion tends to increase. It seems that social and personal variables can be either the antecedents or consequences of self-compassion. Also, a gap underlined in that scoping review were that studies rarely analyzed real-time reactions to failure and stress.

Another literature review on self-compassion and physical activity was conducted by Wong et al. (2021), focusing on self-care and differing from Röthlin et al.’s (2019) study in that it looked at the general population rather than high-level performers. The authors highlighted that self-compassion was a trait allowing a positive and healthy self-care engagement. Self-compassionate individuals were more likely to practice positive thinking, possessed greater self-awareness, and made more accurate self-appraisals. Self-compassion can also be a coping strategy when facing a failure to transform negative or neutral emotions into more positive ones. The authors stated that participating in physical activities was a way to release negative psychological symptoms and improve mental health. Self-compassion seems to enhance people’s physical health and health-related behaviors. Wong et al. (2021) wanted to study the relationship between physical activity and self-compassion. Their main question was: how does physical activity relate to self-compassion? The results’ studies highlighted that self-compassion was associated with self-regulation and thus much likely to process goal settings, behavior evaluation and action-taking. This regulatory behavior, found among highly compassionate people, also applies to evaluations of physical health conditions and yet leading to physical activity-related acts and hence leading to a stronger intrinsic motivation in physical activity engagement (Magnus et al., 2010; Cox et al., 2019), as well as exercise re-engagement after exercise failure or exercise setbacks (Semenchuk et al., 2018).

The present semi-systematic review focuses on the impact and influences between social environments and the psychological competencies of self-compassion and mental toughness within performance settings (sport, music, and dance). It aims to demonstrate the degrees of association and causality between self-compassion, mental toughness, and the social environment within a context of high performance (sport and performing arts).

## Materials and Methods

This semi-systematic review was developed according to the Preferred Reporting Items for Systematic reviews and Meta-Analyses (PRISMA) statement (Moher et al., 2015). An inclusive search strategy was applied, meaning that we sought out both published studies from databases and studies from the gray literature. Review questions were defined, and selection tools were decided before data extraction (inclusion and exclusion criteria). Studies containing the selected keywords were identified *via* a comprehensively search of the following bibliographic databases: APA PsyNet, Renouvaud, ScienceDirect from Elsevier, Pubmed and Google Scholar. A manual search of publication was also done to identify possible missing studies. Studies with relevant titles published in the International Journal of Sport and Exercise Psychology, the Journal of Applied Sport Psychology, Psychology of Sport and Exercise, the Journal on Dance Science, and Psychology of Music were also screened. Additionally, a snowballing technique was adopted to pursue references in referenced articles. The search terms and keywords were developed and checked comparing them with previous literature reviews examining self-compassion, social relationships (interpersonal relationships), and mental toughness. The search terms were: [performance] sports, performing arts, music, dance; [self-compassion] self-kindness, common humanity, mindfulness, isolation, over-identification, self-judgment, self-compassion; [social environment] coach, parents, teammates; [mental toughness] mental toughness. The review identified studies that looked at the relationship between sport or performing arts, self-compassion, social environments, and mental toughness. To ensure that studies’ outcomes were up to date, only relevant studies published since the birth of the concept of self-compassion, in 2003-onwards, were identified. Finally, specific research questions were developed:

1. What associations can be made between mental toughness and self-compassion?
2. What kind of influence do social environments have on the development of self-compassion?

### Inclusion Criteria

**Figure 1:**
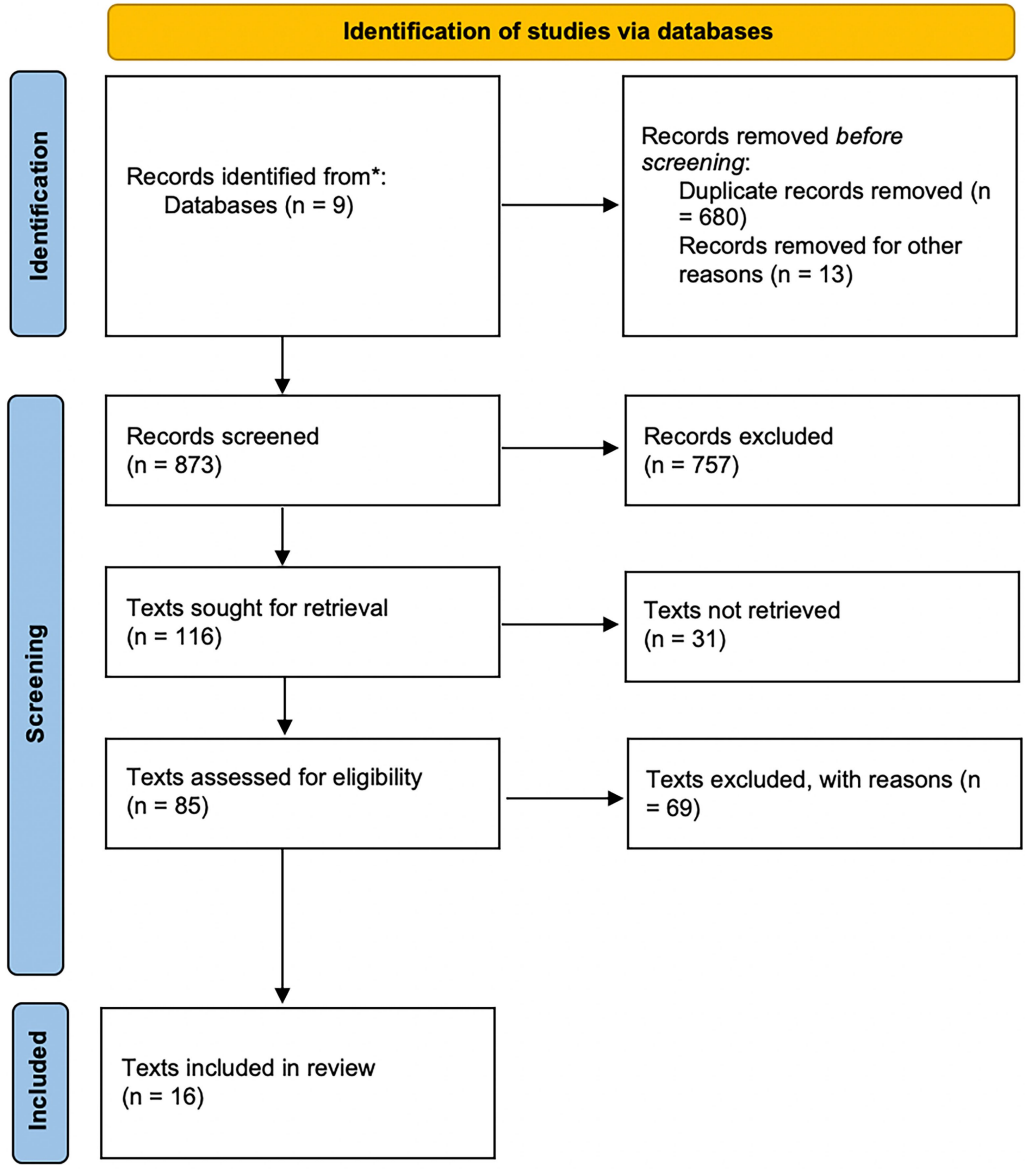
PRISMA diagram of reviewed texts (Page et al., 2021).

No boundaries were set as to the ages of the performers mentioned in the studies reviewed. The study inclusion criteria were as follows: articles and studies written in English or French, including original research or peer-reviewed articles published from 2003 to 2021; examining sport or performing arts settings (music and dance); using qualitative or quantitative methods. Endnote reference management software was used to store and organize all the relevant studies, for screening, and to delete duplicate records. The titles and abstracts of all relevant studies were screened and selected for full-text retrieval and review, based on the inclusion and exclusion criteria. Any study limitations or criticisms that arose on examination, such as sample representativeness or study reliability and validity, were also considered. Articles that did not meet our inclusion criteria were removed from the review process. A flow diagram describing the study identification process is shown in Figure 1.

### Data Extraction

Data from each study included in the review were extracted and coded, including information on the study design and methodology, demographic information including performers’ ages and activity types, as well as the types of instruments used to assess levels of self-compassion, mental toughness, and role or influence of social environments (Table 1). A single investigator extracted the data and screened and reviewed all the papers that met the study’s inclusion criteria.

**Table 1:** Overview of the information retrieved from the abstracts and articles’ description retained for review.

References	Study aims	Sample	Methods
1. Graham-Williams (2013)	Explore transformative changes in self-esteem, self-perception of dance ability, and expressions of spirituality that may occur when adolescent girls not only participate in classical ballet classes but also learn in a teaching model emphasizing compassion, encouragement, discipline, and technique.	27 adolescent girl ballet dancers aged from 14 to 19 years old.	Dancers were divided into 2 groups to compare a traditional, five-week classical ballet class with an experimental class emphasizing compassion, encouragement, discipline, and technique. These attributes were pre- and post-tested using the Rosenberg Self-Esteem Scale (RSES), the dance subscale of Vispoel’s Arts Self-Perception Inventory (ASPI), and the short version of MacDonald’s Expressions of Spirituality Inventory (ESI)
2. Baltzell et al. (2015)	Examine the perceptions of a university women’s Division I soccer coaching staff of their participation with their team in Mindfulness Meditation Training for Sport (MMTS). Report the coaches’ perceptions of the value of the MMTS program to themselves and their athletes, and offer suggestions of how to improve the intervention’s design and delivery.	Three coaches and 19 female soccer players from a Division I varsity women’s soccer team.	Coaches underwent face-to-face interviews after they completed a 6-week, twice-weekly mindfulness and compassion training intervention with their team. Researchers sought to ascertain coaches’ experiences, perceived benefits to their team, and recommendations for improving the design and delivery of the MMTS program.
3. Jeon et al. (2016)	Examine whether self-compassion mediates the relationship between social support and subjective well-being, as perceived by athletes and investigate the structural relationships between these variables.	333 Korean high school or university athletes: 144 university students (123 males, 21 females; mean = 21.5 years, SD = 1.2) and 189 high school students (131 males, 58 females; mean = 17.9 years, SD = 0.8).	Quantitative survey through the completion of the following questionnaires: Self-Compassion Scale, Subjective Well-being Scale, Satisfaction with Life Scale, Korean Emotional Experience Scale, Social Support Scale
4. Fontana et al. (2017)	Examine the relationships between athletes’ perceptions of the motivational climate (caring, task-, and ego-involving) to their levels of compassion, self-compassion, pride, and shame in a recreational sport setting.	164 athletes in a competitive Wiffle Ball tournament.	Quantitative survey through the completion of the following questionnaires: Perceived Motivational Climate in Sports Questionnaire, Caring Climate Scale, the Compassionate Love Scale, the Self-Compassion Scale, the Pride Scale’s Fear of Experiencing Shame and Embarrassment Subscale
5. Tarasoff et al. (2017)	Explore self-compassion in relation to self-evaluative thoughts and behaviors in an evaluative ballet environment.	57 women undergraduate students of whom 30.4% had past dance experience (mean = 20.59 years, SD = 3.81) completed an online questionnaire.	Questionnaires measuring self-compassion, social physique anxiety (trait and state versions), fear of negative evaluation (trait and state versions), as well as reactions, thoughts, and emotions to a hypothetical ‘first day of beginner ballet class’ scenario consistent with the common characteristics of the dance environment.
6. Kveton-Bohnert (2018)	Explore how today’s dancers think about their critical, competitive, and immersive training and how their environment of mirrors, continual teacher correction, and performance demands affects their self-image, emotions, behaviors, and psychological development.	Three age groups of dancers offered narratives: 6 present-day, teenaged, classical ballet students, 6 professional ballet dancers, and 8 adult ballet instructors.	Semi-structured interviews were conducted to gather descriptions of how ballet shapes a dancer’s psyche.
7. Crozier et al. (2019)	Explore the relationship between athletes’ self-compassion and their teammates’ perceived self-compassion.	Team sport competitive athletes (*N* = 108; mean = 23.1 years; SD = 4.68) from soccer (*n* = 12), cricket (*n* = 11), volleyball (*n* = 10), basketball (*n* = 10), ice hockey (*n* = 8), broomball (*n* = 8), wheelchair basketball (*n* = 7), and netball (*n* = 6).	Descriptive norm questions examined participants’ perceptions of teammates’ self-compassion. Three items from the SCS-AV2 scale were modified to reflect perceptions of descriptive norms, targeting athletes’ perceptions of how frequently their teammates acted self-compassionately
8. Hilliard et al. (2019)	Examine the moderating role of self-compassion on the relationship between public stigma and self-stigma, and how self-stigma was associated with attitudes toward seeking counseling.	(*n* = 97 female, *n* = 146 male) representing student athletes from National Collegiate Athletic Association (NCAA) Division I (*n* = 93) and Division III (*n* = 150) institutions. The participants ranged in age from 18 to 23 (M = 19.38, SD = 1.25).	Quantitative survey through the completion of Self-compassion scale, Self-stigma scale, Stigma Scale for Receiving Psychological Help, Attitudes Toward Seeking Professional Psychological Help-Short Form
9. Wilson et al. (2019)	Explore how elite women athletes perceived and experienced mental toughness, self-compassion, and their compatibility in the pursuit of athletic success and stress management.	Seven participants (14 interviews) aged 22–34 (mean = 28.3 years; SD = 5.1), including two half-pipe snowboarders, a swimmer, an ice skater, a downhill mountain-biker, a trampolinist, and a rock climber.	Semi-structured interviews adopting an interpretive, constructionist approach.
10. Kelley and Farley (2019)	Examine how college-age music students report self-compassion in comparison to non-music students.	Participants included 49 music majors and 52 non-music majors (*N* = 101; 56% female).	Two data collections using the 26-item Self-Compassion Scale with an added self-report prompt examining frequency of performance anxiety in the second study.
11. Stamatis et al. (2020)	Investigate self-compassion (SC), mental toughness (MT) and mental health (MH) in a National Collegiate Athletic Association (NCAA) environment for the first time and provide practical suggestions for NCAA MH best practice No.4.	542 student athletes participating across Divisions (mean = 19.84 years; SD = 1.7).	Data were collected using the Mental Toughness Index, the Self-Compassion Scale, and the Mental Health Continuum-Short Form.
12. Johnson (2020)	Explore the relationship between mental toughness and self-compassion in a sport injury context.	Women (*n* = 51) and men (*n* = 30) athletes (Mean = 22.6 years; SD = 4.1) from different sports: ice hockey, soccer, basketball, cross-country, football, gymnastics, rugby, swimming, track and field, volleyball, wrestling.	Mixed methods: Quantitative– currently injured athletes (*n* = 81) completed measures of mental toughness, self-compassion, coping resources, self-esteem, and self-criticism; Qualitative– four semi-structured interviews with athletes from the quantitative study.
13. Frentz et al. (2020)	Explore how high-performance athletes shifted from self-critical to self-compassionate approaches to manage their sporting challenges.	Eleven athletes (6 men, 5 women) aged 19–35 years old (M 1/4 24.7, SD 1/4 4.22), from American football, field hockey, cross-country running, curling, wheelchair rugby, Brazilian jiujitsu, soccer, ice hockey, bobsleigh, swimming, and dance.	One-on-one semi-structured interviews
14. Scott et al. (2021)	Consider how Positive Coaching Alliance (PCA) Triple-Impact Competitor (TIC) workshops could be used to help create positive climates for recreational sport club athletes, and examine how perceptions of a positive team climate related to indices of psychological well-being among sport club athletes.	Recreational college sport club athletes (*N* = 109; 64.2% male; 35.8% female) at a Midwestern university. Mean age = 20.42 years (SD = 1.84).	Facilitating 90-min TIC workshops at the beginning of the fall 2018 season and 30-min follow-up TIC workshops at the beginning of the spring 2019 season for all the officers and coaches of each sport club team. In parallel, conduct a survey including: the Caring Climate Scale; Perceived Motivational Climate Scale; Subjective Happiness Scale; Hope Scale; and Self-compassion Scale
15. Diehl (2021)	Explore how college-student athletes experience and cope with shame-inducing events in their sport and understand the impact of various intrapersonal (self-compassion) and interpersonal (team climate) factors as potential resources or barriers to shame resilience for US college-student athletes.	40 college-student athlete participants (quantitative study) and 15 college-student athlete participants (qualitative study).	Mixed methods: A survey including the Performance Failure Appraisal Inventory’s Fear of Experiencing Shame and Embarrassment subscale, the Perceived Motivational Climate in Sports Questionnaire, the Self-Compassion Scale-Short Form, the Athletic Identity Measurement Scale, Multidimensional Inventory of Perfectionism in Sport, Basic Needs Satisfaction in Sport Scale; There were also semi-structured interviews
16. Maranan and Lopez (2021)	Identify mental skills that aid the development of mental toughness by assessing and exploring the relationships between athletic identity, self-compassion, and intra-team communication.	230 college-student athletes (mean = 20 years; SD = 1; 57% male, *n* = 131) participating in a singles table tennis event.	Multi-part questionnaire using the Athletic Identity Measurement Scale-Plus, Self-compassion Scale (SCS), Scale of Effective Communication in Team Sports (SECTS-2), and the Sports Mental Toughness Questionnaire (SMTQ)

### Outcomes

The primary outcomes were the degrees of association and causality between self-compassion, mental toughness, and the effects of the social environment. Secondary outcomes included the characteristics of the activities examined in the studies reviewed, such as the types of sports practiced, or arts performed.

## Data Synthesis

Our semi-systematic literature review extracted sixteen studies. The main results were synthesized and classified according to the two research questions (Table 2). These results are then discussed in three sections: self-compassion and mental toughness, self-compassion and the social environment, and self-compassion and the performing arts.

**Table 2:** Synthesis of the articles’ aims and results (retrieved from the abstracts) according to the two research questions.

References*	Research question 1. What associations can be made between mental toughness and self-compassion?	Research question 2. What kind of influence do social environments have on the development of self-compassion?
9. Wilson et al. (2019)	Aims: Explore how elite women athletes perceived and experienced mental toughness, self-compassion, and their compatibility in the pursuit of athletic success and stress management.	
	Results: Self-compassion and mindfulness were worthy of investigation among elite women athletes, particularly regarding their utility in coping with sport-related adversity and achieving a mentally tough mindset. Self-compassion and mental toughness are compatible processes that may both require mindfulness and, if used in an effective, complementary balance, could create optimal mindsets for the pursuit of athletic success.
10. Kelley and Farley (2019)	Aims: Examine how college-age music students report self-compassion in comparison to non-music students.	
	Results: The studies revealed no significant difference in self-compassion levels between the groups, although a significant correlation was detected between experiences of performance anxiety and the self-compassion subcomponent of over-identification. Although the results revealed no difference between groups, performance anxiety among musicians may be related to perceived self-compassion.
11. Stamatis et al. (2020)	Aims: Investigate self-compassion (SC), mental toughness (MT) and mental health (MH) in a National Collegiate Athletic Association (NCAA) environment for the first time and provide practical suggestions for NCAA MH best practice No.4.	
	Results: MT, SC (including mindfulness) and MH were positively correlated. Males scored higher than females on all three scales. No differences were found between divisions. SC partially mediated the MT–MH relationship, but moderation was not significant. The authors offered a preliminary step toward promoting resilience and wellness.
12. Johnson (2020)	Aims: Explore the relationship between mental toughness and self-compassion in a sport injury context.	
	Results: Quantitative study findings showed that self-compassion was a significant predictor of mental toughness, coping resources, and self-criticism, beyond the effects of age and self-esteem. Qualitative results described how self-compassion was needed to be mentally tough and that mental toughness was needed to be self-compassionate in sport. They suggested self-compassion was needed to be mentally tough, and vice versa. Future research should examine the relationship between mental toughness and self-compassion over time.
16. Maranan and Lopez (2021)	Aims: Identify mental skills that aid the development of mental toughness by assessing and exploring the relationships between athletic identity, self-compassion, and intra-team communication.	Aims: Identify mental skills that aid the development of mental toughness by assessing and exploring the relationships between athletic identity, self-compassion, and intra-team communication.
	Results: Findings indicated high levels of athletic identity and intra-team communication among the participants, but moderate levels for self-compassion and mental toughness. On one hand, self-compassionate participants who highly recognize their role as athletes, and communicated with their team were mentally tougher. On the other hand, uncompassionate self-responding led student athletes to become mentally weak. The study provided practitioners with useful insights for designing mental skills training geared toward the optimal functioning and psychological wellness of young athletes.	Results: Findings indicated high levels of athletic identity and intra-team communication among the participants, but moderate levels for self-compassion and mental toughness. On one hand, self-compassionate participants who highly recognize their role as athletes, and communicated with their team were mentally tougher. On the other hand, uncompassionate self-responding led student athletes to become mentally weak. The study provided practitioners with useful insights for designing mental skills training geared toward the optimal functioning and psychological wellness of young athletes.
1. Graham-Williams (2013)		Aims: Explore transformative changes in self-esteem, self-perception of dance ability, and expressions of spirituality that may occur when adolescent girls not only participate in classical ballet classes but also learn in a teaching model emphasizing compassion, encouragement, discipline, and technique.
	Results: There was no significant statistical analysis, but the study’s observational findings acknowledged the potential impact of student–teacher rapport in the compassion- and empowerment-based teaching of classical ballet to adolescent girls.
2. Baltzell et al. (2015)		Aims: Examine the perceptions of a university women’s Division I soccer coaching staff of their participation with their team in Mindfulness Meditation Training for Sport (MMTS). Report the coaches’ perceptions of the value of the MMTS program to themselves and their athletes, and offer suggestions of how to improve the intervention’s design and delivery.
	Results: In the main findings, coaches reported experiencing less emotional reactivity to their own negative thoughts and emotions while coaching on the field (at games and practices). They also observed positive changes in how players recovered from mistakes on the field emotionally. Findings suggested that including coaches in mindfulness meditation training programs was beneficial to both coaches and athletes.
3. Jeon et al. (2016)		Aims: Examine whether self-compassion mediates the relationship between social support and subjective well-being, as perceived by athletes and investigate the structural relationships between these variables.
	Results: Self-compassion partially mediated the relationship between social support and subjective well-being. This confirmation demonstrated that self-compassionate attitudes could be fostered by social support, and that, in turn, had a positive effect on an individual’s subjective well-being.
4. Fontana et al. (2017)		Aims: Examine the relationships between athletes’ perceptions of the motivational climate (caring, task-, and ego-involving) to their levels of compassion, self-compassion, pride, and shame in a recreational sport setting.
	Results: Athletes’ perceptions of a caring and task-involving motivational climate were associated with higher levels of compassion for others, but no difference in self-compassion was found. Results suggested that participants in adult recreational sports might benefit from experiencing a positive, supportive team climate.
5. Tarasoff et al. (2017)		Aims: Explore self-compassion in relation to self-evaluative thoughts and behaviors in an evaluative ballet environment.
	Results: Self-compassion was negatively related to trait and state social physique anxiety, trait and state fear of negative evaluation, total negative affect, personalizing thoughts, and catastrophizing thoughts, as well as positively associated with behavioral equanimity. Finding self-compassion to be associated with lower negative self-perceptions within the context of an evaluative beginner ballet class replicated past correlational research and advanced the literature by contextualizing self-compassion to a specific evaluative environment.
6. Kveton-Bohnert (2018)		Aims: Explore how today’s dancers think about their critical, competitive, and immersive training and how their environment of mirrors, continual teacher correction, and performance demands affects their self-image, emotions, behaviors, and psychological development
	Results: The participants indicated that although ballet training was moving away from authoritarian methods, and despite their deep love for the art, they still struggled with negative body image and perfectionist ruminations about flaws. Ballet training should incorporate strategies to build resilience, mindfulness, and self-compassion, which would help dancers develop a healthy inner voice and a growth mindset to sustain them throughout their training and careers. This included fostering self-agency, autonomy, optimism, environmental mastery, personal growth, purpose and meaning, self-acceptance, and positive relations with others. Ways to enhance ballet training to promote intrinsic satisfaction and resilience using mindful learning and a growth mindset were discussed through the lens of positive and humanistic psychology. The goal was to improve dancers’ training to forestall unnecessary suffering and enhance well-being and wholeness.
7. Crozier et al. (2019)		Aims: Explore the relationship between athletes’ self-compassion and their teammates’ perceived self-compassion.
	Results: Athletes’ self-compassion was related to their perceptions of how often their teammates were self-compassionate. Coaches and sport psychologists should encourage athletes to build awareness about how their cognition and behavior relate to others’ cognitions and behaviors.
8. Hilliard et al. (2019)		Aims: Examine the moderating role of self-compassion on the relationship between public stigma and self-stigma, and how self-stigma was associated with attitudes toward seeking counseling.
	Results: Self-compassion was not found to moderate the relationship between public stigma and self-stigma. However, public stigma was positively associated with self-stigma, and self-stigma was negatively associated with attitudes toward counseling. A multigroup analysis found no differences between males and females in this model. The study’s results had implications for professionals working with college-student athletes, suggesting that efforts should aim to reduce stigma and examine alternative factors that might improve attitudes toward seeking help with mental health.
13. Frentz et al. (2020)		Aims: Explore how high-performance athletes shifted from self-critical to self-compassionate approaches to manage their sporting challenges.
	Results: Five themes emerged, covering the key factors in participants’ shifts toward self-compassion: (a) role of the coach, (b) influence of other athletes, (c) impact of important others, (d) developing balanced self-awareness, and (e) maintaining an accepting mindset. Study findings will inform future interventions to support adaptive athlete experiences.
14. Scott et al. (2021)		Aims: Consider how Positive Coaching Alliance (PCA) Triple-Impact Competitor (TIC) workshops could be used to help create positive climates for recreational sport club athletes, and examine how perceptions of a positive team climate related to indices of psychological well-being among sport club athletes.
	Results: Athletes’ perceptions of a caring and task-oriented climate were significantly and positively related to their hope, happiness, and self-kindness. Results suggested that the PCA TIC training was an inexpensive strategy that could foster positive environments within university sport club teams and assist in programs promoting indices of psychological well-being among club sport athletes.
15. Diehl (2021)		Aims: Explore how college-student athletes experience and cope with shame-inducing events in their sport and understand the impact of various intrapersonal (self-compassion) and interpersonal (team climate) factors as potential resources or barriers to shame resilience for US college-student athletes.
	Results: (1) Sport-based shame may negatively impact sport competence and experience; (2) the internalization of the performance ethic (i.e., worth based on outcome success) may lead to sport-based shame; and (3) self-compassion may represent an intrapersonal shame-coping strategy for sport-based shame. In addition, one qualitative-dominant divergent finding revealed that interpersonal support (empathic accuracy, situational feedback, and task/mastery team climates) might lead to intrapersonal shame resilience for college-student athletes.

* *The numbers in front of the references correspond to the numbers in Table 1*.

### Self-Compassion and Mental Toughness

Can mental toughness and inner kindness toward oneself work together to support mental health and performance? Despite differences, it seems that mental toughness and self-compassion share many commonalities (Wilson et al., 2019; Johnson, 2020; Stamatis et al., 2020; Maranan and Lopez, 2021). To the best of our knowledge, Wilson et al. (2019) were the first to explore the compatibility of these two concepts with regards to athletic performance.

Their results suggested that mental toughness and self-compassion were indeed compatible, complementary concepts (Wilson et al., 2019; Johnson, 2020; Stamatis et al., 2020; Maranan and Lopez, 2021). Self-compassion helped performers cope with difficult situations, whereas mental toughness seemed to be helpful in maintaining high motivation, concentration, and resilience (Wilson et al., 2019; Stamatis et al., 2020). Wilson et al. (2019) suggested that if a person cannot move on after facing adversity, they may not be able to maintain an attitude of mental toughness. Therefore self-compassion is crucial to performers.

The participants in the studies investigating these two concepts were mainly women (Wilson et al., 2019; Stamatis et al., 2020). Both quantitative (Stamatis et al., 2020; Maranan and Lopez, 2021) and qualitative (Wilson et al., 2019) methods were used to investigate the relationship between self-compassion and mental toughness. Researchers (Wilson et al., 2019; Johnson, 2020; Stamatis et al., 2020; Maranan and Lopez, 2021) emphasized the importance of working on and developing mental toughness and self-compassion to equip performers to face the challenges they will encounter while allowing them to take care of their mental health. In some athletes, a high level of mental toughness but a low level of self-compassion can lead to difficulties in controlling their emotions in difficult moments (Maranan and Lopez, 2021). Self-compassion can then be useful, filling these gaps by helping these performers to develop an open, understanding attitude toward themselves. Researchers have also looked at parallel concepts such as athletic identity or communication (Maranan and Lopez, 2021).

According to the participants in Wilson et al.’s (2019) study, on the one hand, mental toughness was expressed through perseverance (the capacity to continue despite the difficulties encountered), presence (the capacity to re-focus on what one is doing), perspective-taking and the capacity to prepare oneself for a demanding situation (e.g., competition). On the other hand, despite a lack of understanding of the concept, self-compassion would allow one to develop personal coping resources by feeling that one was not alone and that others sometimes experienced similar situations (i.e., common humanity). In addition, mindfulness—an important aspect of self-compassion— allows performers to keep their attention on the present moment and avoid being caught up in thoughts and emotions (Johnson, 2020; Stamatis et al., 2020; Maranan and Lopez, 2021). Finally, self-compassion was mainly used through positive internal discourse.

Self-compassion seems to help performers develop their mental toughness by allowing them to develop an understanding of what is happening to them, re-evaluate the situation, and then move on (Wilson et al., 2019; Stamatis et al., 2020; Maranan and Lopez, 2021). Some participants also perceived self-compassion as necessary because without it, performers could not be self-aware, supportive, and compassionate enough (with themselves) to deal with the difficult emotions they faced. Self-compassion protected them from the potential pitfalls of a sporting culture of mental toughness that encouraged resilience but emotional avoidance (Johnson, 2020). Self-compassion may lead to mental toughness by developing a better understanding of self-regulation strategies, for example, and thereby help become mentally tough. Moreover, acceptance of injury-related physical limitations (e.g., reduced training intensity and duration) was also important, as it contributed to greater self-awareness. Stamatis et al. (2020) also proposed practical implications. However, there remains a lack of clear guidelines on how athletes, coaches, or parents can proceed to accompany or develop these psychological skills in practice. Skillful use of mental toughness components (e.g., goal commitment, perseverance, task focus) and self-compassion might involve knowing which characteristic to use in a given situation. For example, mental toughness components’ might be useful during competition and self-compassion afterward. The way these psychological processes (e.g., self-compassion, goal setting, perseverance) should be used seems to vary depending on the situation (e.g., during competition, afterward) and the performer itself (e.g., personal characteristics, or need at one specific moment). Anyhow, it seems that these psychological processes are central to become an adaptative and stronger performer, but more research is needed to understand how and when to exercise these different skills.

Mindfulness is also a key link between mental toughness and self-compassion. Wilson et al.’s (2019) and Johnson’s (2020) participants used it to regulate attention and emotion (two key dimensions of Gucciardi’s concept of mental toughness). This may also support the notion that mindfulness should be studied further as a possible component of not only self-compassion but also mental toughness (e.g., a possible additional key dimension in Gucciardi’s one-dimensional concept of mental toughness).

### Self-Compassion and the Social Environment

Our literature review found that the environment in which performers practiced had a crucial impact and influence on the development of self-compassion (Graham-Williams, 2013; Fontana et al., 2017). A performer’s social environment is often created by their coach as well as by their peers and parents (Frentz et al., 2020). The coach appears to have a crucial role to play in the development of skill of self-compassion (Frentz et al., 2020). Fontana et al. (2017) highlighted the link between perceptions that an environment was caring and the level compassion one might feel for peers, teammates, or a coach. For others, compassion seems to be influenced by a caring environment (Fontana et al., 2017; Crozier et al., 2019). Some researchers have also highlighted the negative correlation between ego-centric environments and how forgiving one might be toward teammates who make mistakes or failure (Fontana et al., 2017). In contrast, the link between self-compassion and a caring atmosphere is not as clear (Fontana et al., 2017; Hilliard et al., 2019). Some studies have highlighted the need to develop a self-compassion tool specific to the world of performers because, to date, Neff’s (2003b) scale to detect self-compassion in the general population might be interpreted by them as self-pity, depending on the items (Fontana et al., 2017).

Some studies have focused on implementing intervention programs to help coaches and/or performers develop skills in mindfulness and self-compassion (Baltzell et al., 2015; Scott et al., 2021). Data have highlighted that these different types of themes could have impacts on coaches: increased sensitivity to their athletes’ feelings, increased knowledge and awareness of their own feelings, and improved coach–athlete relationships (Baltzell et al., 2015; Frentz et al., 2020), as well as increased skills in self-compassion and well-being (Scott et al., 2021). Athletes appear to be more effective at recovering emotionally after a difficult situation and feel more care and warmth for themselves and their peers. Creating compassionate environments seems to enable athletes to create better adaptation mechanisms (Scott et al., 2021).

Studies looking at self-compassion or its development through the creation of a compassionate environment have mainly focused on performers’ perspectives (Fontana et al., 2017). Coaches, as well as the performers circle of acquaintances (e.g., parents, peers), have been studied less (Baltzell et al., 2015; Frentz et al., 2020) and it would be interesting to investigate coaches’ influence and ability to develop compassionate environments for performers’ performance and well-being. Social support and the creation of supportive and compassionate environments do, however, appear to effect self-compassion (Graham-Williams, 2013; Jeon et al., 2016), despite the mixed results revealed by Fontana et al. (2017). Social support and the creation of supportive environments can improve a performer’s coping skills and contribute to more positive emotions and greater perceived self-worth (Graham-Williams, 2013; Jeon et al., 2016; Crozier et al., 2019; Frentz et al., 2020; Diehl, 2021). Individuals’ attitudes toward themselves are influenced by the evaluations of significant others.

Studies with athletes show that the feeling of social support (Jeon et al., 2016) or perceiving teammates to be highly self-compassionate (Crozier et al., 2019) increases well-being. Other situational features, such as the motivational atmosphere, are not correlated with self-compassion (Fontana et al., 2017). Thus, studies investigating self-compassion should consider these aspects to be potential moderators (e.g., whether athletes in teams demonstrating little self-compassion, great concerns about perfectionism, or low levels of social support would benefit markedly from greater self-compassion).

### Self-Compassion and the Performing Arts

To date, some research on self-compassion has been done among dancers (Graham-Williams, 2013; Ingstrup et al., 2017; Tarasoff et al., 2017; Kveton-Bohnert, 2018). However, to the best of our knowledge, only one study focusing on self-compassion has been carried out in the music field (Kelley and Farley, 2019). It is, therefore, important to consider these few studies considering broader research in the field of performance psychology. These performers face many challenges—as do athletes—that need to be overcome and learning environments are likely to be a key location where performers can be equipped with skills such as self-compassion and mental toughness.

Research in the field of the performing arts has focused primarily on dance, highlighting the deleterious role that their working environments sometimes have on dancers’ well-being and level of self-compassion (Graham-Williams, 2013; Tarasoff et al., 2017). Researchers point out that the environments in which dancers practice and perform subject them to a form of constant evaluation that is not conducive to looking at oneself with compassion and kindness, leading to detrimental effects on one’s self-image (Kveton-Bohnert, 2018). Researchers have provided evidence that a musician or dancer’s level of self-compassion can be improved using therapeutic means that can lead to better mental health outcomes (Tarasoff et al., 2017; Kveton-Bohnert, 2018; Kelley and Farley, 2019). The results of these studies were like those exploring self-esteem in the performing arts or self-compassion among athletes. Graham-Williams (2013) looked at dance education methods and their influence on dancers’ levels of self-compassion. The results were not significant but teaching with a focus on compassion and caring nevertheless seemed to have beneficial effects on those performers’ well-being.

The field of performing arts is still under-researched. It would seem important, when studying self-compassion and mental toughness, to include musicians, dancers, and performers to create a bridge between the worlds of sport and the performance arts, which are not so different from each other in many respects.

## Discussion

This semi-systematic literature review examined the relationships between self-compassion, mental toughness, and the influence of social environments on performers (athletes, dancers, and musicians). The review showed that self-compassion was positively correlated with social support and was a good complement to mental toughness. But it also showed that there was a lack of knowledge about this concept in the field of the performing arts (dance and music). Moreover, the results highlighted the importance of looking at why self-compassion does or does not develop and, more importantly, how to help performers and those around them develop it.

Most of the studies covered in this review examined the potential benefits of self-compassion on well-being and social relationships. Few, however, examined the influence of self-compassion on sports and artistic performance. Developing studies highlighting the links between self-compassion and performance could improve acceptance of ideas about this concept among these populations.

The literature reviewed showed that the positive relationships between self-compassion, mental toughness, and social environments exists in populations of athletes (Fontana et al., 2017; Wilson et al., 2019; Frentz et al., 2020; Johnson, 2020; Stamatis et al., 2020; Maranan and Lopez, 2021). Studies suggest that self-compassion is a psychological resource that should be developed alongside mental toughness and that the use of either concept depends on the context and setting. Self-compassion and mental toughness are related but distinct concepts, and they have been insufficiently studied together from the perspective of mental health or performance in sport and the arts. Both help to enhance performance and well-being by supporting performers as they attempt to manage stress, regulate thoughts and emotions, focus and re-focus their attention, and persevere despite setbacks.

The interacting effects of mental toughness and self-compassion on mental health are, therefore, an unresolved issue that deserves further investigation. Are they buffering, reinforcing, or antagonistic effects? Optimal mental health in sport may not only be about mental toughness and self-compassion, but also about the timing and context of their application. One hypothesis would be to adapt the concept of self-compassion to the world of sport by competency through mental toughness, two concepts to be studied in parallel (Adam et al., 2021). Self-compassion would also help performers overcome feelings of stigmatization over their vulnerabilities by allowing them to seek help. The research reviewed showed that more self-compassionate athletes reported seeking the help they thought necessary more frequently than their less self-compassionate peers (Scott et al., 2021). Future studies should include more representative samples from a wider range of performers (i.e., athletes, dancers, and musicians) to investigate the potential effects of different performance cultures.

This review sought to provide an overview of the current literature on self-compassion, mental toughness, and the social environment in the worlds of sport (athletes) and performing arts (dancers and musicians). We conclude that the number of multi-day intervention programs and longitudinal studies should be increased. These studies should consider assessing the specific aspects of performance culture and settings that could alter the effectiveness of these interventions. In addition, overall performance specific measures could be developed to assess general levels of self-compassion. The development of a theoretical framework explaining how self-compassion affects a performer, the role of their entourage and its link to other psychological resources, such as mental toughness, could help to better understand this concept. The development of self-compassion has also proven to be an important driver in the search for psychological help (Heath et al., 2017) in the general population. Indeed, it could be hypothesized that developing a compassionate and caring environment around performers would help them to develop self-compassion which, as shown in various research studies, improves well-being and performance (Jeon et al., 2016; Frentz et al., 2020; Scott et al., 2021). It could also lead to performers being more open to seeking psychological help when needed (Adam et al., 2021). This might make it possible to better prevent certain psychological shifts or difficulties (e.g., doubting their personal capabilities, feeling bad about themselves) that performers currently keep silent for fear of appearing weak.

It is also important to emphasize the practical implications of applying self-compassion to the worlds of sport and the performing arts. Based on the current literature, self-compassion seems to be particularly useful when trying to increase performers’ well-being or help them better deal with stress, failure, and setbacks. Athletes’ skepticism about self-compassion should also be taken seriously. Interventions intended for performers should first check whether participants have concerns about the concept, explain clearly why self-compassion has the potential to improve performance (e.g., by helping athletes deal with failure), demonstrate the links between self-compassion and mental toughness, and acknowledge that current research is still ongoing on how to apply self-compassion in performance contexts. Moreover, further research should be carried out to design interventions adapted to performers’ entourages (i.e., coaches, parents), stakeholders who play crucial roles in the development of psychological skills such as self-compassion and mental toughness.

## Author Contributions

CK, RAP, and VP contributed to the conception and the design of the study. CK organized the database, performed the analysis, and wrote the first draft of the manuscript. CK and RAP contributed to manuscript revision. All authors contributed to the article and approved the submitted version.

## Funding

Open access funding provided by University of Lausanne.

## Conflict of Interest

The authors declare that the research was conducted in the absence of any commercial or financial relationships that could be construed as a potential conflict of interest.

## Publisher’s Note

All claims expressed in this article are solely those of the authors and do not necessarily represent those of their affiliated organizations, or those of the publisher, the editors and the reviewers. Any product that may be evaluated in this article, or claim that may be made by its manufacturer, is not guaranteed or endorsed by the publisher.
